# Habitat divergence shapes the morphological diversity of larval insects: insights from scorpionflies

**DOI:** 10.1038/s41598-019-49211-z

**Published:** 2019-09-03

**Authors:** Lu Jiang, Yuan Hua, Gui-Lin Hu, Bao-Zhen Hua

**Affiliations:** 1 Key Laboratory of Plant Protection Resources and Pest Management, Ministry of Education, College of Plant Protection, Northwest A&F University, Yangling, Shaanxi 712100 China; 2 Key Laboratory of Economic and Applied Entomology of Liaoning Province, College of Plant Protection, Shenyang Agricultural University, Shenyang, Liaoning 110866 China; 3 College of Life Sciences, Northwest A&F University, Yangling, Shaanxi 712100 China

**Keywords:** Biodiversity, Electron microscopy, Entomology

## Abstract

Insects are the most diverse group of organisms in the world, but how this diversity was achieved is still a disputable and unsatisfactorily resolved issue. In this paper, we investigated the correlations of habitat preferences and morphological traits in larval Panorpidae in the phylogenetic context to unravel the driving forces underlying the evolution of morphological traits. The results show that most anatomical features are shared by monophyletic groups and are synapomorphies. However, the phenotypes of body colorations are shared by paraphyletic assemblages, implying that they are adaptive characters. The larvae of *Dicerapanorpa* and *Cerapanorpa* are epedaphic and are darkish dorsally as camouflage, and possess well-developed locomotory appendages as adaptations likely to avoid potential predators. On the contrary, the larvae of *Neopanorpa* are euedaphic and are pale on their trunks, with shallow furrows, reduced antennae, shortened setae, flattened compound eyes on the head capsules, and short dorsal processes on the trunk. All these characters appear to be adaptations for the larvae to inhabit the soil. We suggest that habitat divergence has driven the morphological diversity between the epedaphic and euedaphic larvae, and may be partly responsible for the divergence of major clades within the Panorpidae.

## Introduction

Insects are the most diverse organisms on the earth, exhibiting the most diverse morphological features and occupying a wide range of ecological niches^1,2^. The driving forces leading to this diversity have long received great attention from evolutionary biologists. Some authors consider that the diversity is attributed to co-radiation with angiosperm plants, judged from evidence of herbivorous or pollinating insects^3–7^. Others argue that it is correlated with complicated sexual behaviors, focusing on the sexually mature adults^8,9^. With respect to the insects that neither feed on plants nor undergo sexual reproduction, however, the driving force of diversity is still disputable.

Larvae, the immature stage of holometabolous insects, represent a significant developmental stage in insects’ life history^2^, and are considered feeding devices that acquire nutrition for the development of pupal and imaginal stages^10,11^. On the other hand, larvae as a free-living life stage also have their own survival requirements and often exhibit dramatically diverse morphological features as well as biological characteristics^12,13^. This morphological diversity is not only distinct among distant groups, but also may be noticeable in closely related species^14^. However, the information on insect larvae is only available for about 2% of known holometabolous species^15,16^. Our knowledge of larvae is even more limited, fragmentary to be more specific, for the groups of less economic significance as in Mecoptera.

The larvae of Mecoptera exhibit a wide range of morphological diversity^17–19^, although Mecoptera, as a small order, are only composed of nine extant families with approximately 650 extant species in the world^20–22^. The larvae of Nannochoristidae are campodeiform and aquatic, living in the substrate of streams^23–25^. The larvae of Boreidae are scarabaeiform and herbivorous, creeping on or boring into live mosses^26–28^. In most other families of Pistillifera (including Apteropanorpidae, Choristidae, Bittacidae, Panorpodidae, and Panorpidae), the larvae are generally eruciform and edaphic, occurring in the soil or on the ground^18,29,30^ and exhibiting different feeding habits at the family level^31^.

Panorpidae is the largest family in Mecoptera and comprises approximately 470 species currently assigned to eight genera, mainly confined to the Northern Hemisphere^32–37^. The eruciform larvae of Panorpidae inhabit the soil^38–43^. Based on our recent investigations, the larvae of different panorpid species exhibit notable morphological differences on head capsules, thoracic legs, abdominal prolegs, spiracles, and chaetotaxy^44–47^. However, whether these morphological and biological differences reflect the evolutionary process or are just adaptations for habitats has not been clarified to date.

In this study, eight species in four genera of Panorpidae were investigated and compared for larval morphological traits and correlated habitat preferences. Both morphological and habitat features were marked on a molecular phylogenetic tree in order to understand further how this larval morphological diversity was achieved.

## Methods

### Insect collection and rearing

Live adults were captured with sweeping nets in Shaanxi Province, central China from early June to late August in 2015. Detailed collection information is listed in Table 1.

**Table 1:** Information of studied organisms and their localities in Shaanxi, China.

Taxa	Localities	Dates	Collectors
*Dicerapanorpa magna* (Chou in Chou *et al*., 1981)	Tiantaishan (34°13′N, 106°59′E)	vi. 2015	Lu Jiang
*Dicerapanorpa* sp.	Hualongshan (32°01′N, 109°21′E)	vii. 2015	Lu Jiang
*Cerapanorpa nanwutaina* (Chou in Chou *et al*., 1981)	Tongtianhe (34°11′N, 106°40′E)	vi. 2015	Lu Jiang
*C. dubia* (Chou & Wang in Chou *et al*., 1981)	Tiantaishan (34°13′N, 106°59′E)	vi. 2015	Lu Jiang
*Panorpa curva* Carpenter, 1938	Tongtianhe (34°11′N, 106°40′E)	vi. 2015	Lu Jiang
*P. chengi* Chou in Chou *et al*., 1981	Huoditang (33°26′N, 108°27′E)	viii. 2015	Jie Lu & Lu Liu
*Neopanorpa lipingensis* Cai & Hua, 2009	Zhenping (31°53′N, 109°31′E)	vii. 2015	Lu Jiang
*N. longiprocessa* Hua & Chou, 1997	Huoditang (33°26′N, 108°27′E)	vii. 2015	Jie Lu & Lu Liu

To rear the scorpionflies, loamy soil was collected from the Tiantaishan Forest Park in the Qinling Mountains, where adult scorpionflies were collected, as rearing substrate. The soil was kept in a sealed valve bag, sterilized in an autoclave at 85 °C for 30 min, left to cool for 3 h, and then sprayed little water till the moisture reached approximately 15% (M/M).

Adults were reared in pairs in plastic jars covered with gauze under natural conditions. The jars were filled with 4–5 cm moist loamy soil in depth. Both adults and larvae were daily provided chopped mealworms as food items^48^. Live fourth-instar larvae (within 5 d after molting) were used for behavioral experiments. Photographs were taken with a Nikon D90 digital camera (Nikon, Tokyo, Japan).

### Morphological observations

For morphological observations, the larvae were fixed in Carnoy’s solution for 12 h and preserved in 75% ethanol. The fixed larvae were dissected and dehydrated in a graded ethanol series, replaced by tertiary butanol, freeze-dried for 3 h, sputter-coated with gold, and examined under a Hitachi S-3400N scanning electron microscope (Hitachi, Tokyo, Japan) at 5 kV.  Morphological graphpads were provided in the supplementary dataset 2.

### Habitat preferences

To examine habitat preferences under laboratory conditions ((19 ± 1) °C, 75% ± 5% relative humidity, 14L:10D photoperiod), fourth-instar larvae (*n* = 50) were placed on the soil surface (7 cm in depth) in a plastic box (25 cm × 15 cm × 15 cm) at daytime. Numbers of larvae remained on the soil surface were recorded successively in the following three hours. For each species the experiment was replicated three times.

To investigate circadian rhythmicity, fourth-instar larvae (*n* = 50) were reared under the laboratory condition mentioned above. The numbers of larvae on the soil were recorded every two hours over a period of 24 h. The experiment was replicated three times in three successive days for each species. Data analyses were carried out using R ver. 3.3.1 (R Development Core Team, Vienna, Austria). Original data of the behavior experiments were provided in the supplementary dataset 1.

### Molecular phylogenetic analyses

Two mitochondrial gene fragments (cytochrome *c* oxidase subunits I and II, *COI* and *COII*) and one nuclear gene fragment (28S ribosomal RNA, 28*S rRNA*) were used in the phylogenetic analyses. The DNA sequences of *Dicerapanorpa* sp. and *Neopanorpa lipingensis* were generated based on primers from previous studies^49,50^. Other DNA sequence data were obtained from a previous study^33^. Two species of Panorpodidae, *Panorpodes kuandianensis* and *Brachypanorpa carolinensis*, were used as outgroups.

DNA sequences were checked, assembled and edited with SeqMan^51^. Multiple sequence alignment was performed using ClustalX 2.0.21 with default parameters^52^. The gappy regions at the beginning and end of the alignment were manually deleted with BioEdit 7.0.9.0^53^. The best partition schemes and models were estimated for the whole data matrix based on the program PartitionFinder v1.1.1^54^ under Bayesian Information Criterion (BIC). The concatenated dataset was divided into four partitions, first codon position of *COI* and *COII* with TrN + I, second codon position of *COI* and *COII* with HKY + I, third codon position of *COI* and *COII* with HKY + G, and *28S* with HKY + G.

### Ethics approval and consent to participate

No specific permits were required for the described field studies: a) no specific permissions were required for the locations/activities; b) locations were not privately owned or protected; and c) the field studies did not involve endangered or protected species.

## Results

### Morphological comparison

**Figure 1:**
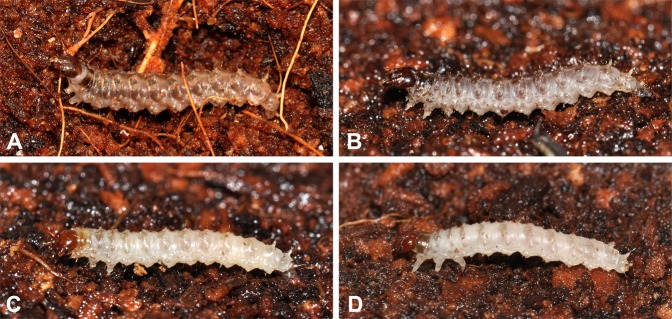
Larvae of Panorpidae in habitus. (**A**) *Dicerapanorpa* *magna*; (**B**) *Cerapanorpa* *nanwutaina*; (**C**) *Panorpa* *curva*; (**D**) *Neopanorpa* *lipingensis*.

The larvae of all panorpid species are eruciform, possessing three pairs of thoracic legs and eight pairs of abdominal prolegs (Fig. 1). The larval heads are heavily sclerotized, bearing a pair of compound eyes, a pair of 3-segmented antennae, and the mandibulate mouthparts that are directed ventrally. The larval trunks are roughly cylindrical, equipped with paired erect subdorsal annulated processes on A1–A9 and a single mid-dorsal annulated process on A10. The paired dorsal processes on A1–A7 are much shorter than those on A8–A10. The body color varies dorsally among the genera.

The larvae of *Dicerapanorpa* are darkish brown dorsally and possess two dark broad subdorsal stripes (Fig. 1A). The larvae of *Cerapanorpa* are darkish grey dorsally, but dull pale ventrally (Fig. 1B). The larvae of *Panorpa* and *Neopanorpa* are grossly pale on the whole body, including dorsal and ventral surfaces (Fig. 1C,D).

**Figure 2:**
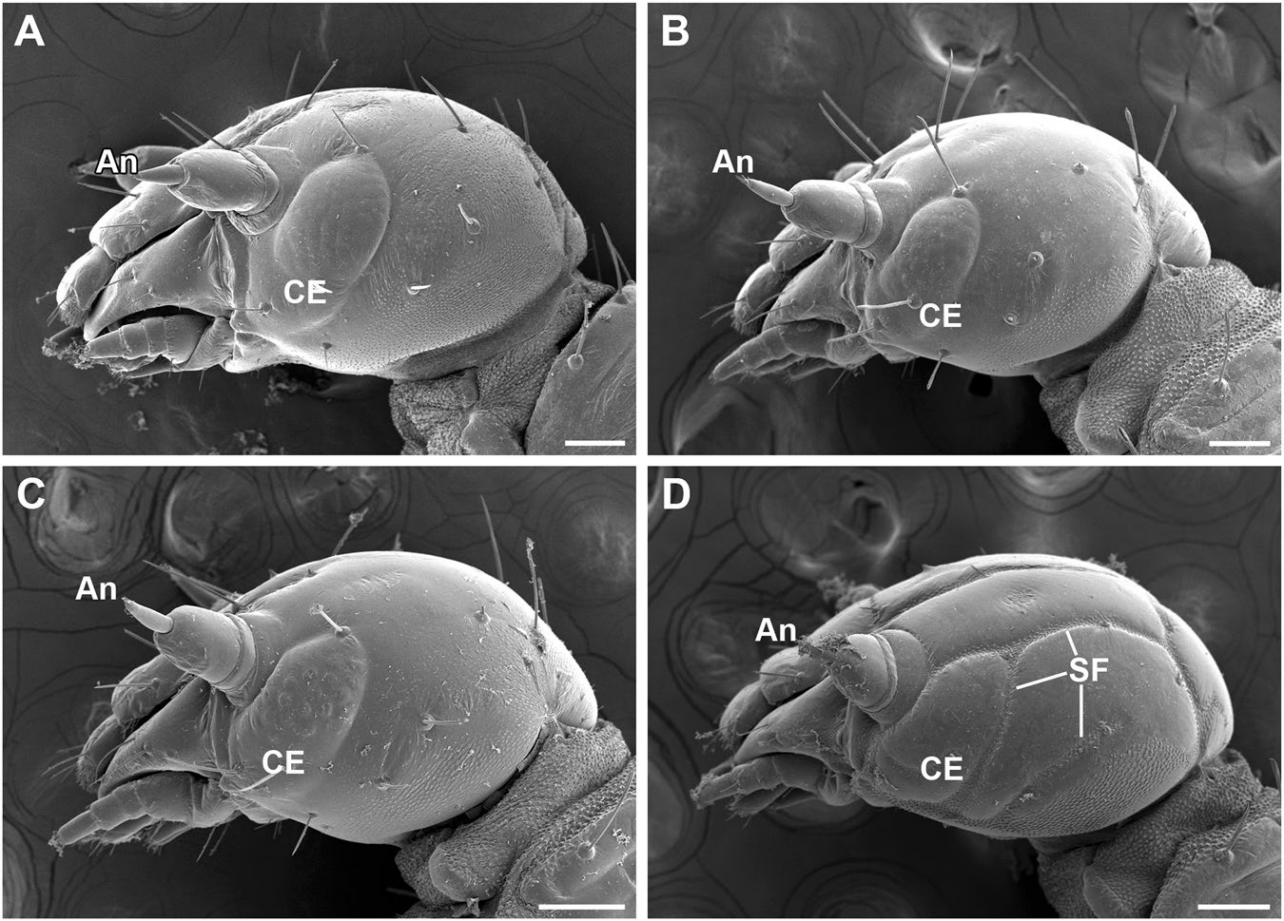
Larval heads of Panorpidae (lateral views). (**A**) *Dicerapanorpa magna*; (**B**) *Cerapanorpa nanwutaina*; (**C**) *Panorpa curva*; (**D**) *Neopanorpa lipingensis*. An, antenna; CE, compound eye; SF, shallow furrow. Scale bars = 100 μm.

The head capsules are furnished with 13 pairs of setae arranged symmetrically. The setae vary in length among the species and are much shorter in *Neopanorpa* than in other three genera (Fig. 2).

The head capsules are delimited by several normal sutures and sulci in all the species (Fig. 2A–C), but some distinct shallow furrows are present in the occipital, coronal and genal areas in *Neopanorpa* larvae (Fig. 2D).

The larval compound eyes are externally protuberant in *Dicerapanorpa*, *Cerapanorpa*, and *Panorpa* with clearly defined facets of ommatidia (Fig. 2A–C), but roughly flat in *Neopanorpa* with the facets of ommatidia indistinct (Fig. 2D).

The three-segmented antennae are slender and longer in *Dicerapanorpa*, *Cerapanorpa*, and *Panorpa* than in *Neopanorpa* (Fig. 2).

**Figure 3:**
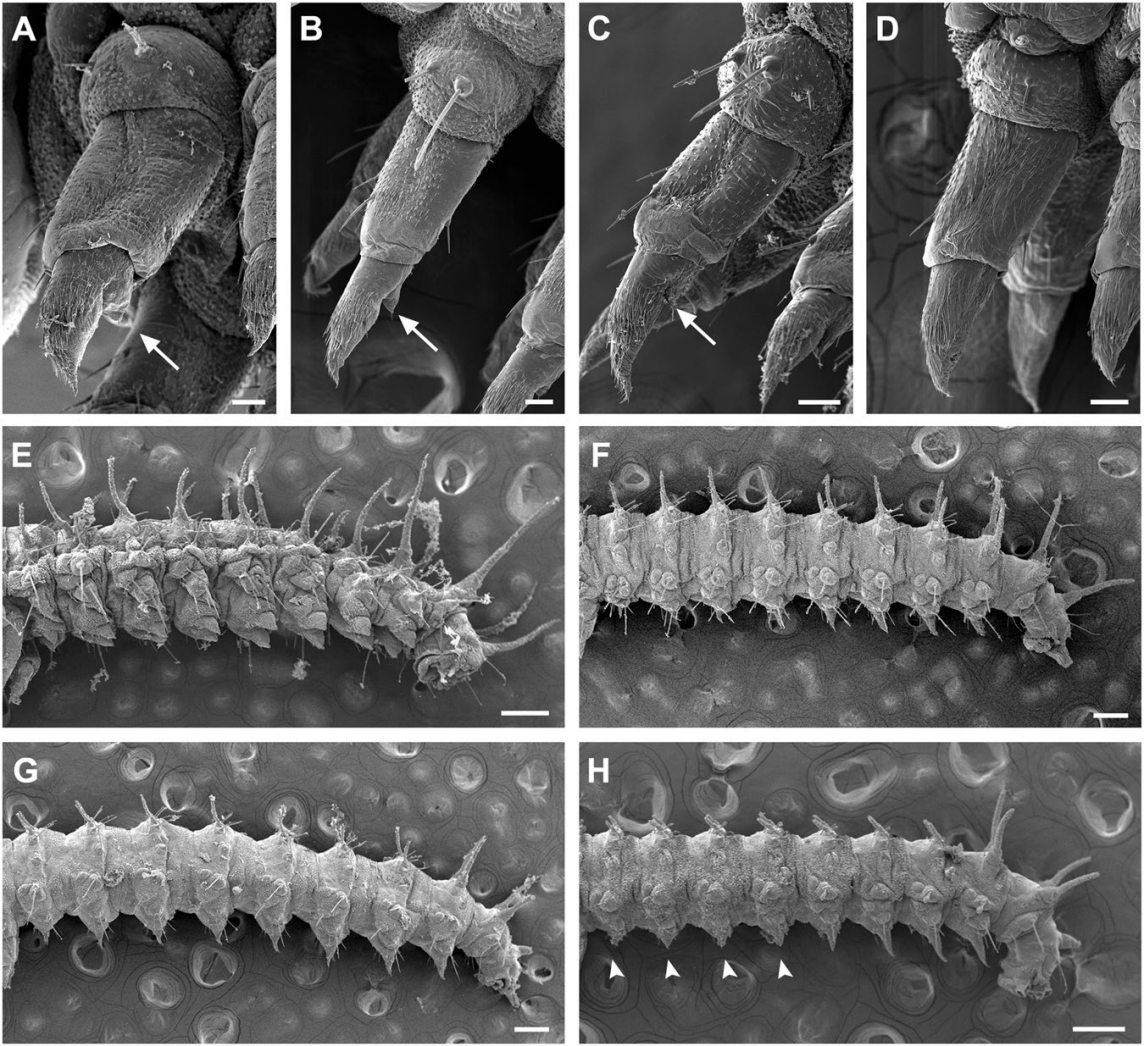
Larval thoracic legs and prolegs of Panorpidae. (**A**) and (**E**) *Dicerapanorpa magna*; (**B**) and (**F**) *Cerapanorpa nanwutaina*; (**C**) and (**G**) *Panorpa curva*; (**D**) and (**H**) *Neopanorpa lipingensis*; (**A**–**D**) prothoracic legs, arrows point to tibial lobes; (**E–H**) prolegs, arrow heads point to morphologically reduced prolegs on the anterior four abdominal segments. Scale bars: (**A**–**D**) = 40 μm; (**E**)–(**H**) = 200 μm.

The thoracic legs are four-segmented, each consisting of a stout coxa, a cylindrical elongate femur, a slender tibia, and a short pointed hirsute tarsus. A remarkable triangular tibial lobe is present posteromesally on the tibiae of *Dicerapanorpa*, *Cerapanorpa*, and *Panorpa* (Fig. 3A–C), but absent in *Neopanorpa* (Fig. 3D).

Hirsute unsegmented ventral prolegs are present on the first eight abdominal segments. These prolegs are well developed in *Dicerapanorpa*, *Cerapanorpa*, and *Panorpa* (Fig. 3E–G), but remarkably reduced on the anterior four pairs in *Neopanorpa* (Fig. 3H).

The dorsal annulated processes are very similar between the species of each genus, but vary in length among the genera. These dorsal processes are very prominent in *Dicerapanorpa*, median-sized in *Cerapanorpa*, and relatively shorter in *Panorpa* and *Neopanorpa* (Fig. 3).

### Habitat preference

**Figure 4:**
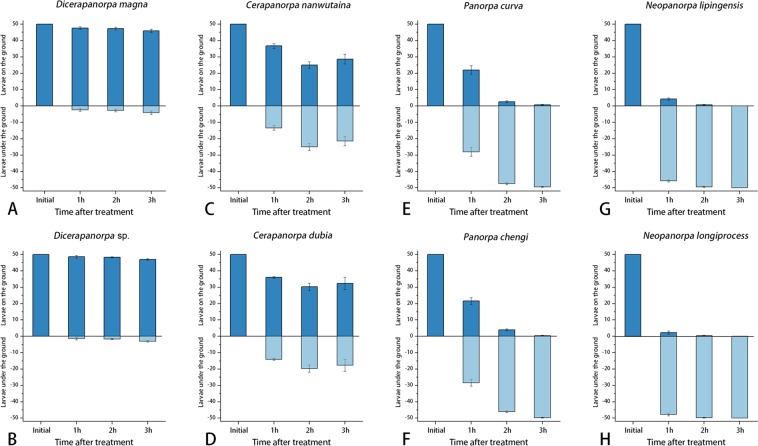
The number of larvae on/under the ground in three successive hours (Mean ± SEM, *n* = 3), showing the habitat preference of larvae. (**A**) *Dicerapanorpa magna*; (**B**) *Dicerapanorpa* sp.; (**C**) *Cerapanorpa nanwutaina*; (**D**) *Cerapanorpa dubia*; (**E**) *Panorpa curva*; (**F**) *Panorpa chengi*; (**G**) *Neopanorpa lipingensis*; (**H**) *Neopanorpa longiprocessa*.

Larval habitat preferences were very similar between the species of each genus, but varied significantly among the genera (Fig. 4).

In *Dicerapanorpa* more than 45 of 50 individuals stayed consistently on the soil (Fig. 4A,B), indicating that the larvae of *Dicerapanorpa* are epedaphic.

In *Cerapanorpa* the number of larvae on the soil decreased immediately, but then remained at around half number (Fig. 4C,D). This suggests that the larvae of *Cerapanorpa* are hemi-epedaphic, occurring either on the ground or shallowly in the soil.

In *Panorpa* over half individuals entered the soil within 1 h exposure to light, and almost all the larvae burrowed into the soil in 3 h (Fig. 4E,F), indicating that the larvae of *Panorpa* are euedaphic, preferring to inhabit the soil.

In *Neopanorpa* the larvae immediately burrowed into the soil and became completely invisible in 3 h (Fig. 4G,H), showing that the larvae of *Neopanorpa* are also euedaphic, inhabiting the soil most of their life time.

### Circadian rhythms

**Figure 5:**
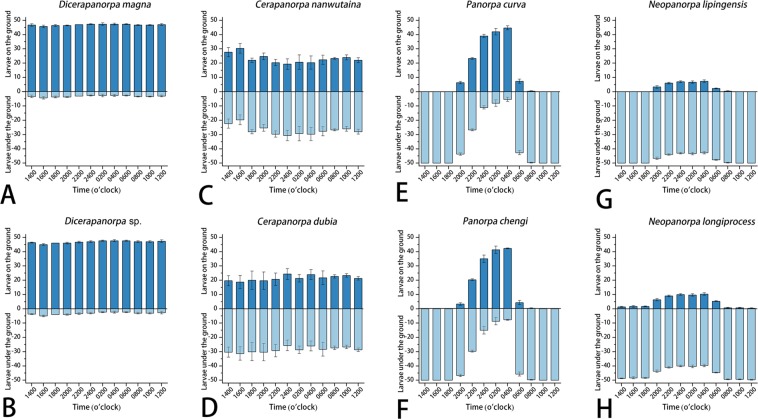
The number of larvae on/under the ground every two hours in a day (Mean ± SEM, *n* = 3), showing the circadian rhythm of larvae. (**A**) *Dicerapanorpa magna*; (**B**) *Dicerapanorpa* sp.; (**C**) *Cerapanorpa nanwutaina*; (**D**) *Cerapanorpa dubia*; (**E**) *Panorpa curva*; (**F**) *Panorpa chengi*; (**G**) *Neopanorpa lipingensis*; (**H**) *Neopanorpa longiprocessa*.

The larval circadian rhythms also exhibited very similar patterns between the species of each genus, but were significantly different among the four genera (Fig. 5).

In *Dicerapanorpa* at any time of the day or night, the number of larvae on the soil amounted to at least 45 of 50 (Fig. 5A,B), indicating that the larvae of *Dicerapanorpa* exhibit no behavioral difference during the whole day.

In *Cerapanorpa* nearly half number of larvae stayed on the soil the whole day, with the number fluctuating slightly between 20 and 30 (Fig. 5C,D), also showing no distinct behavioral difference.

The larvae of *Panorpa* exhibited remarkable differences between the day and night because they were invisible during the day from 0800 to 1800, but present on the soil surface after 2000 or 2200, before burrowing into the soil again when the sun rose at 0600 or 0800 (Fig. 5E,F). This indicates that the larvae of *Panorpa* are nocturnal, mainly active during the night.

In *Neopanorpa* the number of larvae on the soil fluctuated only slightly between the day and night. Approximately 10 of 50 larvae stayed on the soil at night from 2000 to 0600, but very few of them were visible on the soil during the day from 0800 to 1800 (Fig. 5G,H). This further indicates that the larvae of *Neopanorpa* are euedaphic and nocturnal, most of which inhabit the soil during the day, and only comparatively active at night.

### Molecular phylogeny and character evolution

**Figure 6:**
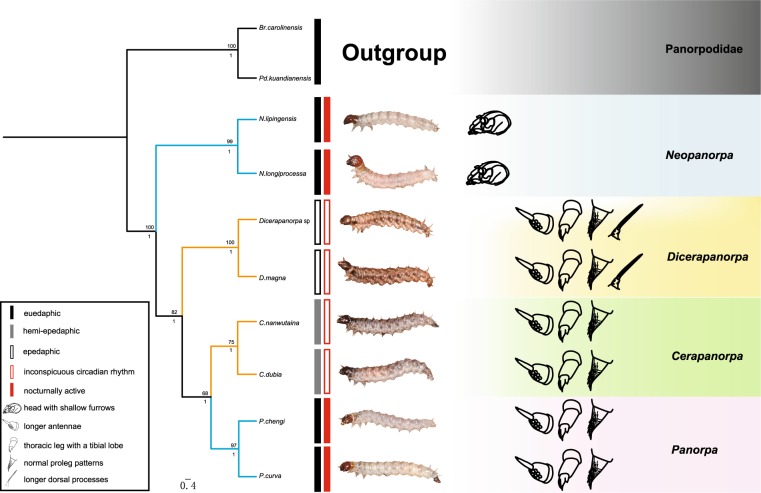
Maximum likelihood chronogram of the eight panorpid species inferred from the concatenated *COI*, *COII* and *28S*. Node numbers above nodes show bootstrap values, numbers below node show posterior probability. The vertical bars in the middle depict the larval habits of species. The right markings show the morphological features of panorpid larvae.

Bayesian and maximum likelihood methods yielded phylogenetic trees of similar topologies in the combined three-gene analysis. Here, the consensus tree from the ML analysis was adopted to summarize the results (Fig. 6). All the four genera were confirmed to be monophyletic, with a high support value (posterior probability = 1). *Neopanorpa* diverged from other genera the earliest in the panorpid species studied. *Dicerapanorpa* is the sister group to *Cerapanorpa* + *Panorpa*.

In general, morphological characters of larvae evolved congruently with the tree topology. The presence of shallow furrows on the larval head is definitely a synapomorphy supporting the monophyly of *Neopanorpa*. The longer antennae, presence of tibial lobe on thoracic legs, and proleg patterns strongly support the monophyly of *Dicerapanorpa* + (*Cerapanorpa* + *Panorpa*) (Fig. 6). On the contrary, the phenetic similarities with respect to body coloration are divergent from the tree topology. The darkened color of larval body is shared by the paraphyletic grade of *Dicerapanorpa* + *Cerapanorpa*. The pale color of body is shared by the assemblage of *Neopanorpa* + *Panorpa* (Fig. 6).

## Discussion

Theoretically, the phenetic similarities could be considered as synapomorphies or homoplasy, depending on whether the species assemblage constitute a monophyletic group^55^. In this study, most anatomical characters of larvae are synapomorphies supporting the monophyly of Panorpidae. However, the phenetic similarities of body coloration are shared by paraphyletic assemblages, and are highly related to the specific habitats, implying the adaptive evolution of these edaphic larvae.

Edaphic arthropods can be further subdivided into epedaphic (living on the soil) and euedaphic (inhabiting the soil), and have evolved a range of morphological, physiological and behavioral characteristics that work in concert to permit survival in different microhabitats^56^. Epedaphic insects, like other exposed insects, need a variety of adaptive strategies, including efficient locomotive abilities^57^, well-developed visual organs^58^, and various kinds of protective body coloration^59^. Euedaphic insects, on the contrary, often bear fossorial legs or sclerotized mandibles for digging and burrowing in the substrate^56,60^, and smaller body size to pass unrestrictedly through the pre-existing tunnels in the soils^61,62^. Based on our present investigation, both epedaphic and euedaphic adaptive strategies were found in the larvae of Panorpidae.

The larvae of *Dicerapanorpa* are generally epedaphic, preferring to stay on the soil in the day and night. The larvae of *Dicerapanorpa* with a darkish dorsum have the color of soil for camouflage, as discussed in our previous study^46^. This strategy is also adopted by the ground-crawling larvae of Bittacidae, which spray soil on their body surface as camouflage^48,63–65^. Moreover, the larvae of *Dicerapanorpa*, *Cerapanorpa*, and *Panorpa* permanently or temporarily stay on the soil, all possessing well-developed thoracic legs with tibial lobes and well-developed abdominal prolegs (Fig. 6). These structures are likely to promote above-ground locomotive abilities and to support the larval abdomen during crawling on the soil^66^.

The larvae of *Neopanorpa* are exclusively euedaphic. These larvae prefer to stay in the soil all the time during the day and night. The euedaphic larvae of *Neopanorpa* all have shallow furrows on their head capsules^41,67^. The furrows help withstanding considerable mechanical pressure imposed on the head capsules during burrowing^46^. In addition, the antennae, cephalic setae, dorsal processes on the trunk, and four anterior pairs of prolegs are all shorter in the euedaphic larvae of *Neopanorpa*. These should facilitate their movement through the soil by decreasing friction and resistance^61,62^. It is worth noting further that the larval compound eyes of *Neopanorpa* are externally flat, and appears to be another adaptation to reduce friction.

With regard to habitat divergence, panorpid larvae are not only divided spatially, but also separated temporally. Nocturnal habit has evolved in the larvae of *P. curva*, *P. chengi*, *N. lipingensis*, and *N. longiprocessa*, similar to *P. liui*, *P. virginica* and *P. lugubris*, whose “above-ground searching” behaviors usually occur in the dark^38,45,46^. The nocturnal activity of these larvae likely reduces predation risk, as the night may offer safer foraging opportunities, i.e. protection from visually hunting predators^68^. Because they never expose to the light on the soil, it is unnecessary for the larvae of *Panorpa* and *Neopanorpa* to adopt camouflage strategies. As a result, these larvae are grossly pale, similar to soil-inhabiting white grubs of Scarabaeoidea^69–71^ and stem-boring weevils of Curculionidae in Coleoptera^72^.

Differing from herbivores or sexually mature insects, the saprophagous larvae of Panorpidae have no peculiar requirements for the co-evolution with specific host plants or heterosexual individuals^10,12,19^. The diversifying driving force of these edaphic saprophagous larvae probably attributes to the competition of habitat in the soil, which provide them organic matters as food^31^ and cavities as living space^46^. Owing to competition, the sympatric panorpid larvae that share a common ancestor switched to divergent ecological niches by subdividing the soil microhabitats on spatial and temporal aspects. In divergent niches, the larvae employ appropriate adaptive strategies that reflected diverse morphological features involving head capsules, setae, prolegs, and dorsal processes. On the other hand, the congeneric larvae occupying similar habitats share a common adaptive strategy and thus also morphological features. Consequently, our results would suggest that the habitat divergence has driven the morphological diversity, and may be partly responsible for the divergence of Panorpidae.

## Supplementary information

Supplementary Dataset 1

Supplementary Dataset 2
