# Supplementary material for: Habitat divergence shapes the morphological diversity of larval insects: insights from scorpionflies
Source: Sci Rep. 2019 Sep 3;9:12708. doi: 10.1038/s41598-019-49211-z (PMC6722236; doi:10.1038/s41598-019-49211-z)
Supplement: Supplementary file 1 — Supplementary Dataset 1 [file 41598_2019_49211_MOESM1_ESM.pdf]

Supplementary information 1 (Original data of the behavior experiments)

Habitat divergence shapes the morphological diversity of larval insects: insights from scorpionflies

Lu Jiang[1,2†], Yuan Hua[1,3†], Gui-Lin Hu[1], and Bao-Zhen Hua[1,*]

[1]*Key Laboratory of Plant Protection Resources and Pest Management, Ministry of Education, College of Plant Protection, Northwest A&F University, Yangling, Shaanxi 712100, China.*

[2]*Key Laboratory of Economic and Applied Entomology of Liaoning Province, College of Plant Protection, Shenyang Agricultural University, Shenyang, Liaoning 110866, China.*

[3]*College of Life Sciences, Northwest A&F University, Yangling, Shaanxi 712100, China.*

[†]*These authors contributed equally in this work.*

[*]*Correspondence author (E-mail: huabzh@nwafu.edu.cn)*

Original data of the experiment 1 (Habitat preference)

Dicerapanorpa magna

Initial	1h	2h	3h
50	48	46	47
50	49	49	47
50	46	47	44

Dicerapanorpa sp.

Initial	1h	2h	3h
50	50	48	47
50	49	48	48
50	47	49	46

Cerapanorpa nanwutaina

Initial	1h	2h	3h
50	39	28	24
50	34	26	34
50	37	21	28

Cerapanorpa dubia

Initial	1h	2h	3h
50	36	33	36
50	37	32	36
50	35	26	25

Panorpa curva

Initial	1h	2h	3h
50	27	2	1
50	18	4	1
50	21	2	0

Panorpa chengi

Initial	1h	2h	3h
50	25	5	0
50	22	4	1
50	18	3	0

Neopanorpa lipingensis

Initial	1h	2h	3h
50	5	1	0
50	3	0	0
50	5	1	0

Neopanorpa longiprocess

Initial	1h	2h	3h
50	2	0	0
50	4	1	0
50	1	0	0

Original data of the experiment 2 (Circadian rhythms)

Dicerapanorpa magna

1400	1600	1800	2000	2200	2400	0200	0400	0600	0800	1000	1200
48	47	47	47	47	47	46	48	47	47	47	48
47	46	47	46	47	48	49	48	48	47	47	47
45	44	45	46	47	47	47	46	47	46	46	46

Dicerapanorpa sp.

1400	1600	1800	2000	2200	2400	0200	0400	0600	0800	1000	1200
47	46	46	46	46	46	47	49	48	48	48	49
46	45	46	45	46	47	48	47	47	46	46	46
46	44	46	47	48	48	48	47	48	47	47	47

Cerapanorpa nanwutaina

1400	1600	1800	2000	2200	2400	0200	0400	0600	0800	1000	1200
34	37	24	27	22	23	23	21	22	24	24	22
24	26	19	20	16	12	11	12	17	24	27	25
25	28	23	27	23	23	28	28	28	22	21	19

Cerapanorpa dubia

1400	1600	1800	2000	2200	2400	0200	0400	0600	0800	1000	1200
19	20	20	18	21	24	21	24	19	21	21	19
14	10	9	10	13	18	17	18	15	22	25	23
26	26	31	31	28	31	26	30	31	25	24	22

Panorpa curva

1400	1600	1800	2000	2200	2400	0200	0400	0600	0800	1000	1200

0	0	0	6	22	41	46	45	10	1	0	0
0	0	0	8	24	37	42	47	7	0	0	0
0	0	0	5	24	39	38	42	5	0	0	0

Panorpa chengi

1400	1600	1800	2000	2200	2400	0200	0400	0600	0800	1000	1200
0	0	0	3	19	30	37	43	7	1	0	0
0	0	0	5	21	39	46	42	4	0	0	0
0	0	0	2	21	36	41	42	2	0	0	0

Neopanorpa lipingensis

1400	1600	1800	2000	2200	2400	0200	0400	0600	0800	1000	1200
0	0	0	3	5	7	5	6	3	0	0	0
0	0	0	2	7	8	8	7	2	0	0	0
0	0	0	5	6	6	7	9	2	1	0	0

Neopanorpa longiprocess

1400	1600	1800	2000	2200	2400	0200	0400	0600	0800	1000	1200
1	1	2	6	8	10	8	9	6	1	1	1
2	3	1	5	10	11	11	10	5	0	1	0
1	1	2	8	9	9	10	12	5	1	0	0
