# Supplementary material for: Habitat divergence shapes the morphological diversity of larval insects: insights from scorpionflies
Source: Sci Rep. 2019 Sep 3;9:12708. doi: 10.1038/s41598-019-49211-z (PMC6722236; doi:10.1038/s41598-019-49211-z)
Supplement: Supplementary file 2 — Supplementary Dataset 2 [file 41598_2019_49211_MOESM2_ESM.pdf]

Supplementary information 2 (Morphological graphpads of the scorpionfly larvae)

Habitat divergence shapes the morphological diversity of larval insects: insights from scorpionflies

Lu Jiang[1,2†], Yuan Hua[1,3†], Gui-Lin Hu[1], and Bao-Zhen Hua[1,*]

[1]*Key Laboratory of Plant Protection Resources and Pest Management, Ministry of Education, College of Plant Protection, Northwest A&F University, Yangling, Shaanxi 712100, China.*

[2]*Key Laboratory of Economic and Applied Entomology of Liaoning Province, College of Plant Protection, Shenyang Agricultural University, Shenyang, Liaoning 110866, China.*

[3]*College of Life Sciences, Northwest A&F University, Yangling, Shaanxi 712100, China.*

[†]*These authors contributed equally in this work.*

[*]*Correspondence author (E-mail: huabzh@nwafu.edu.cn)*

Dicerapanorpa magna

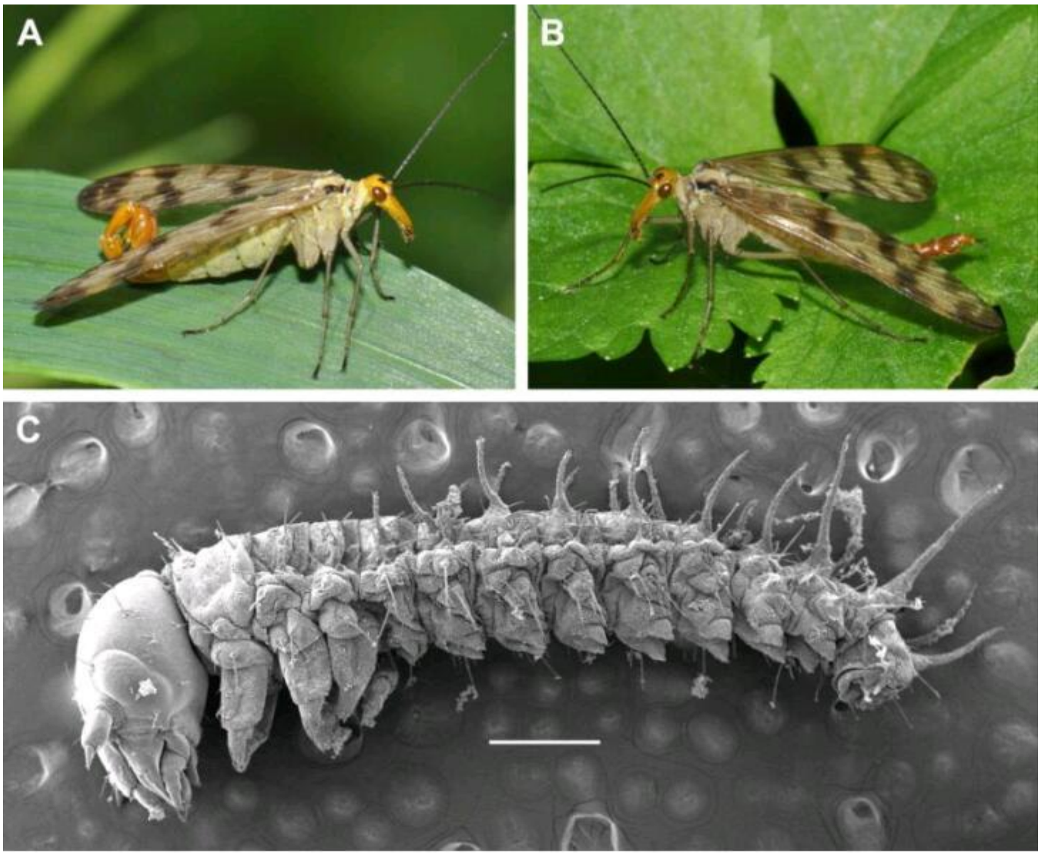

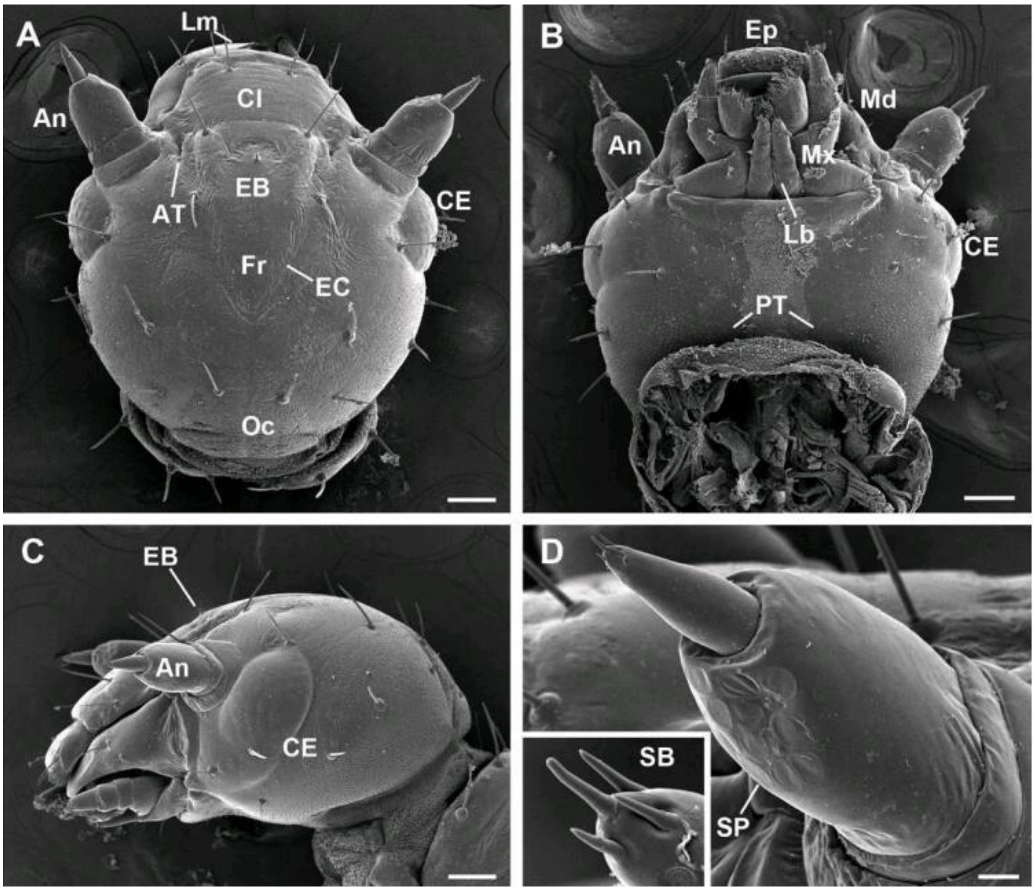

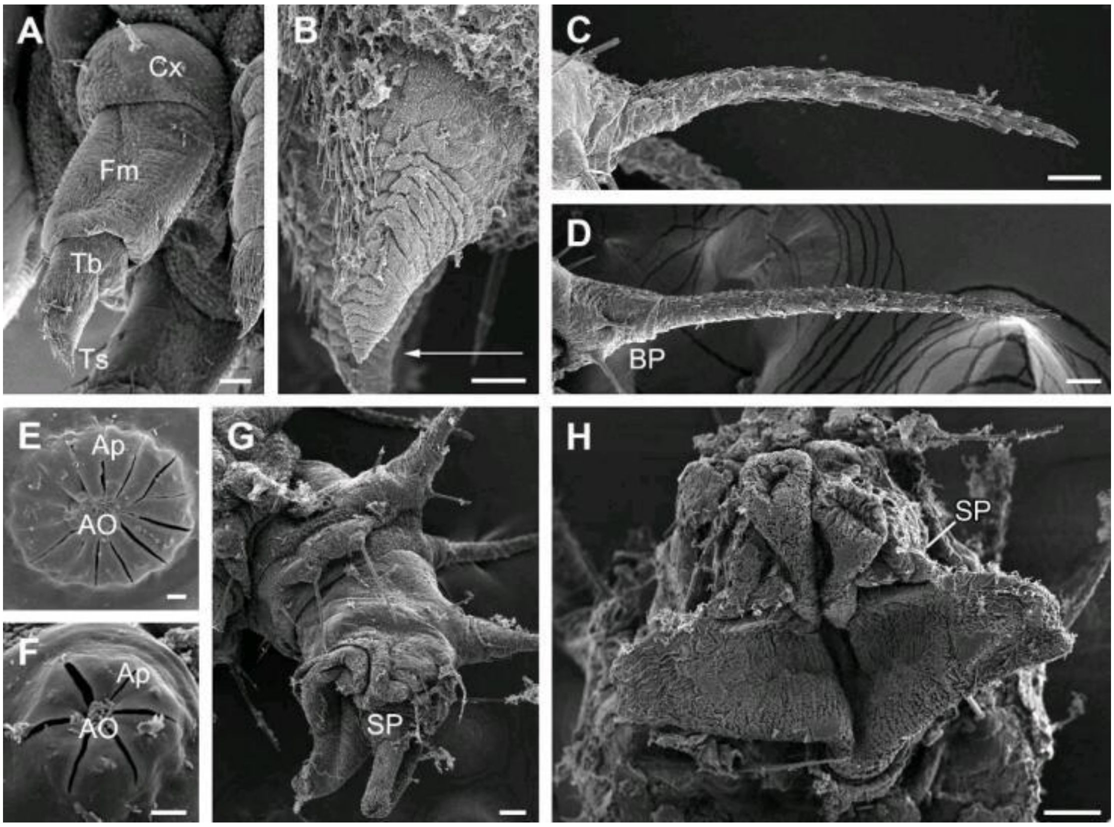
A
Cx
Fm
Tb
Ts
B
C
D
BP
E
Ap
AO
F
Ap
AO
G
SP
H
SP

Dicerapanorpa sp.

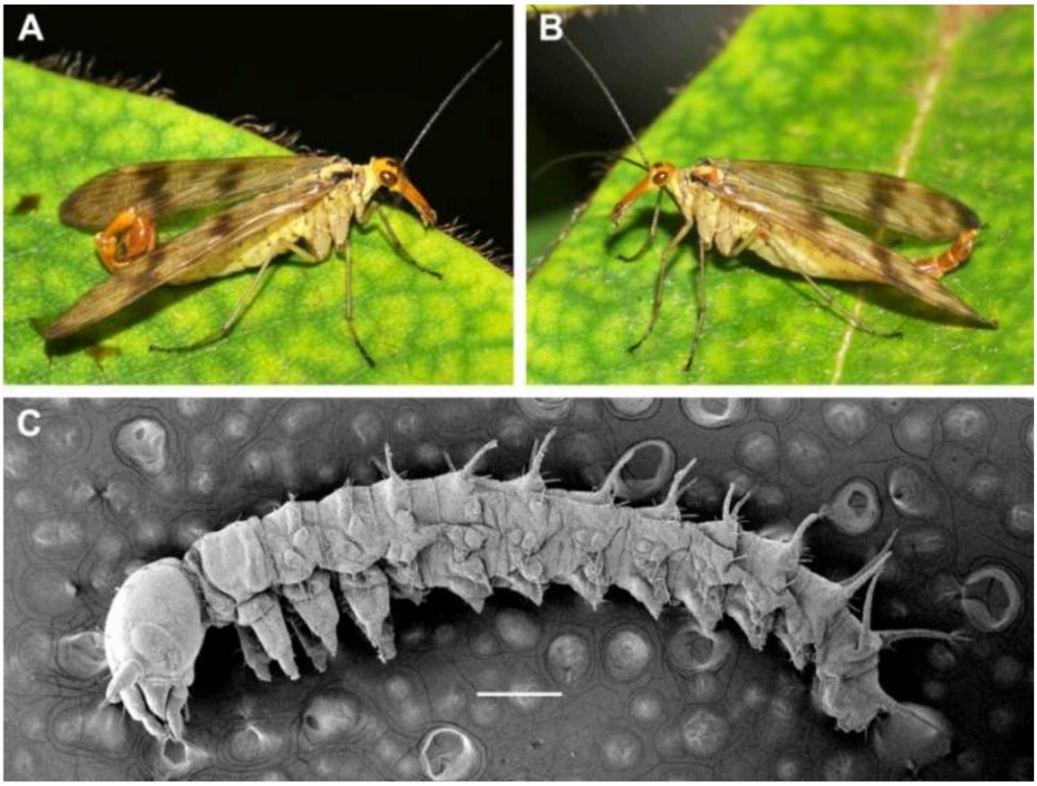

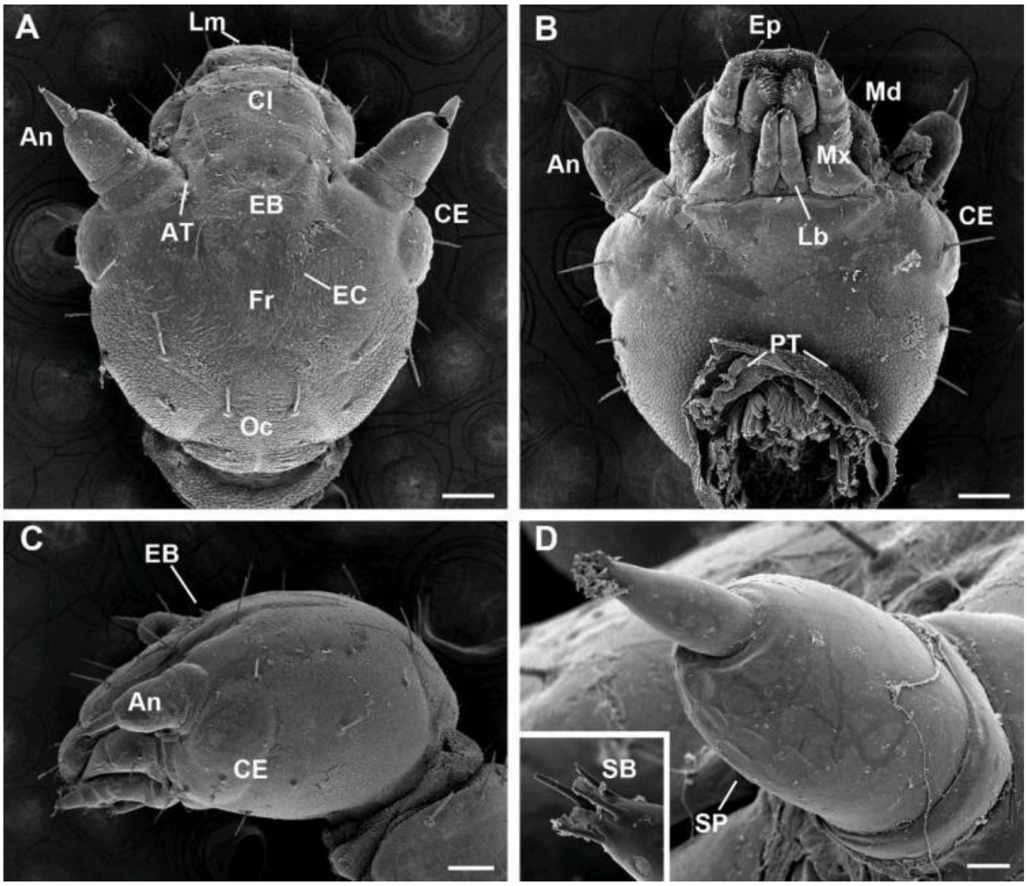

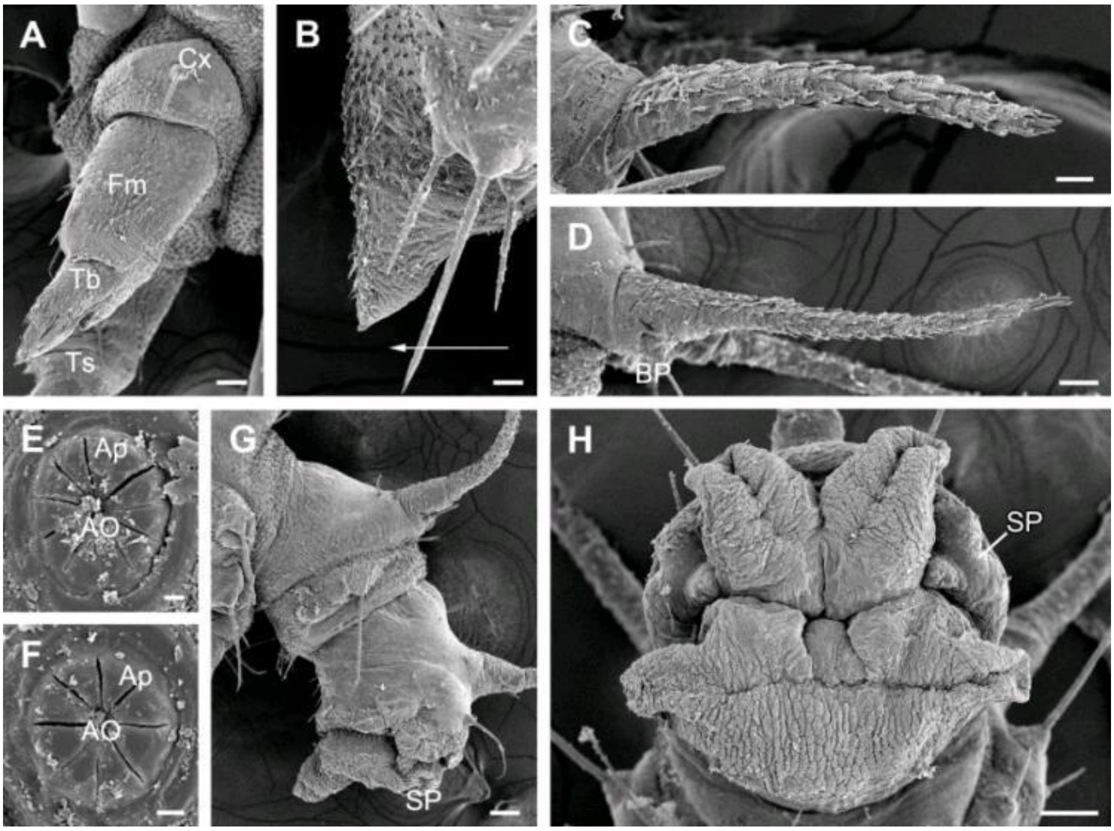
A
Cx
Fm
Tb
Ts
B
C
D
BP
E
Ap
AO
F
Ap
AO
G
SP
H
SP

Cerapanorpa nanwutaina

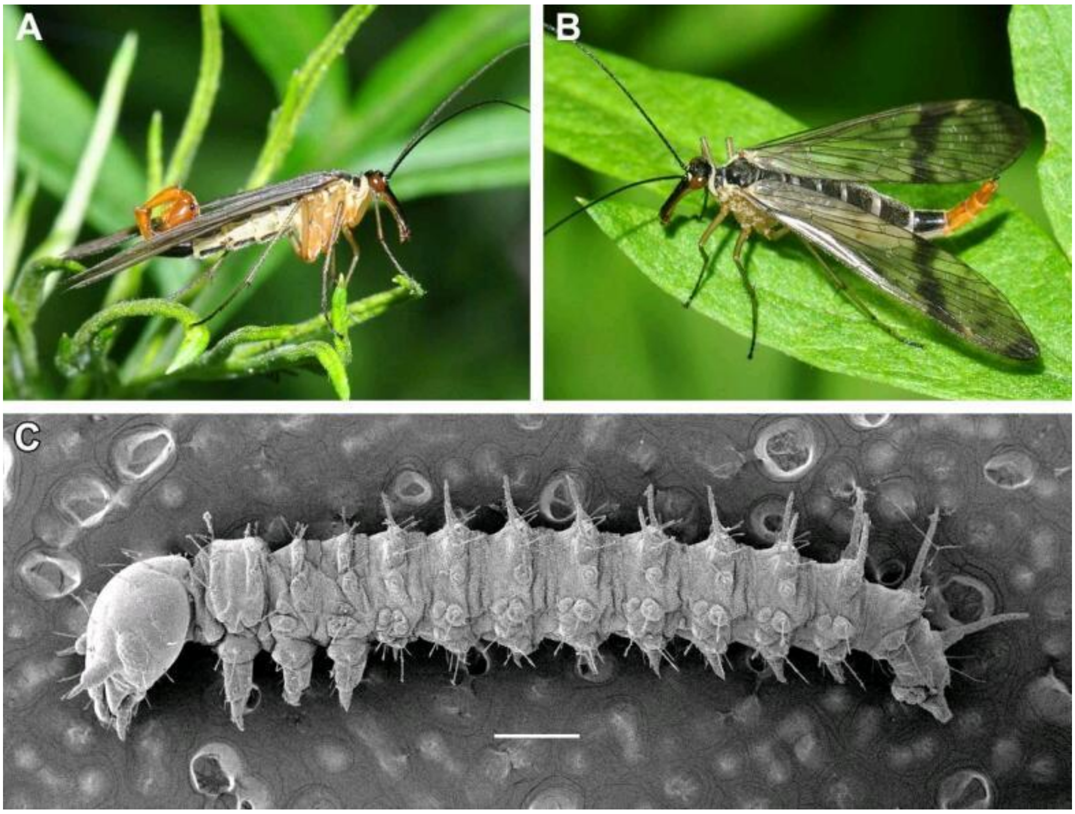

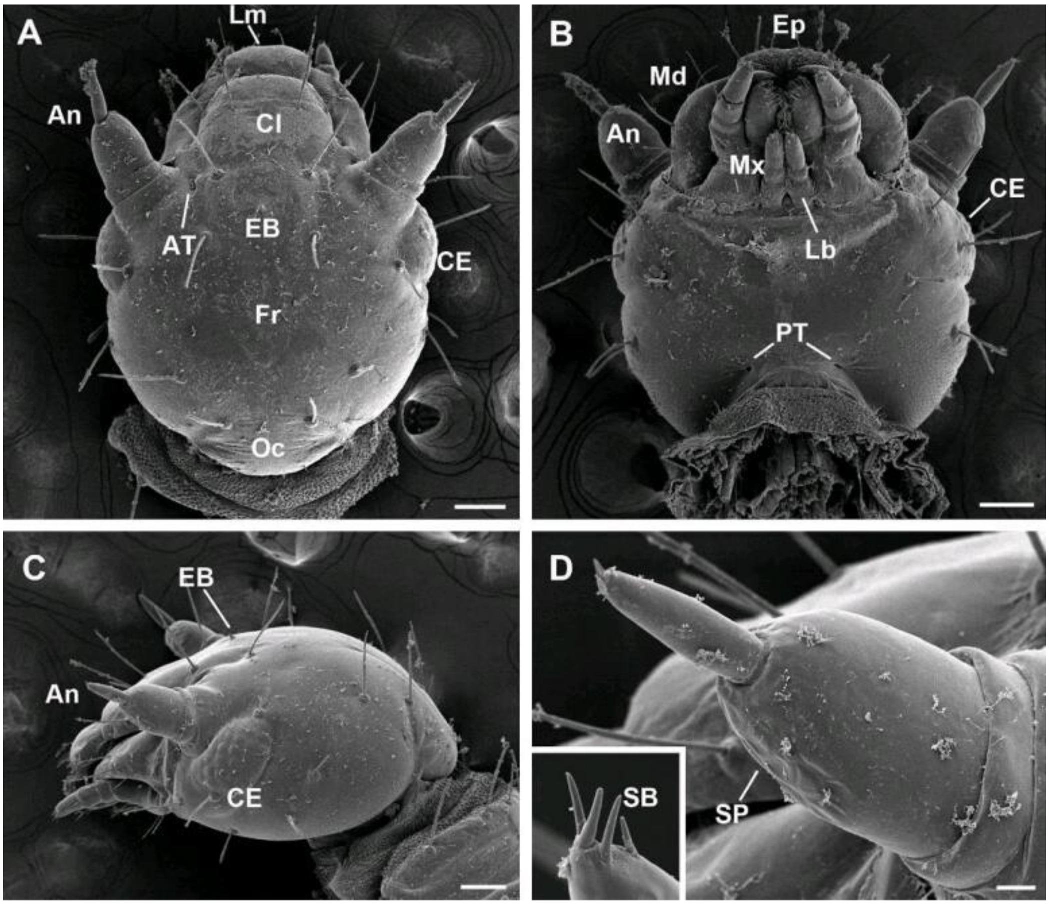

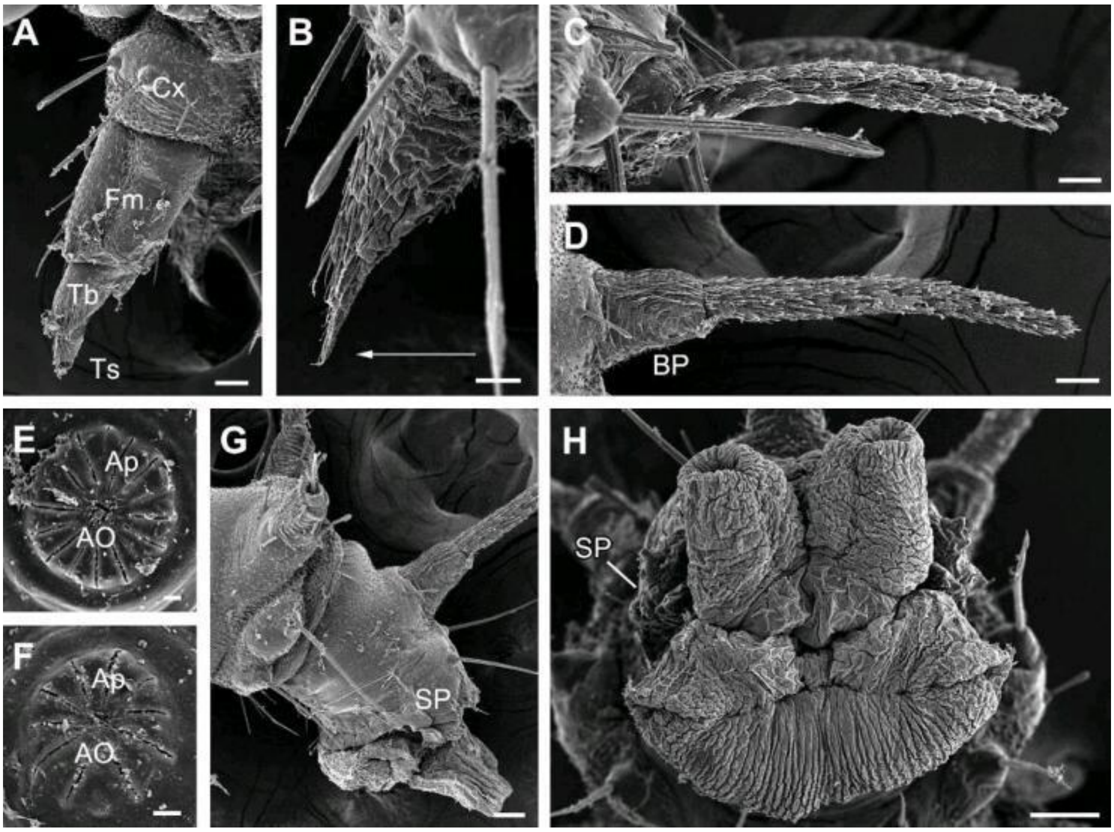

A
Cx
Fm
Tb
Ts
B
C
D
BP
E
Ap
AO
F
Ap
AO
G
SP
H
SP

Cerapanorpa dubia

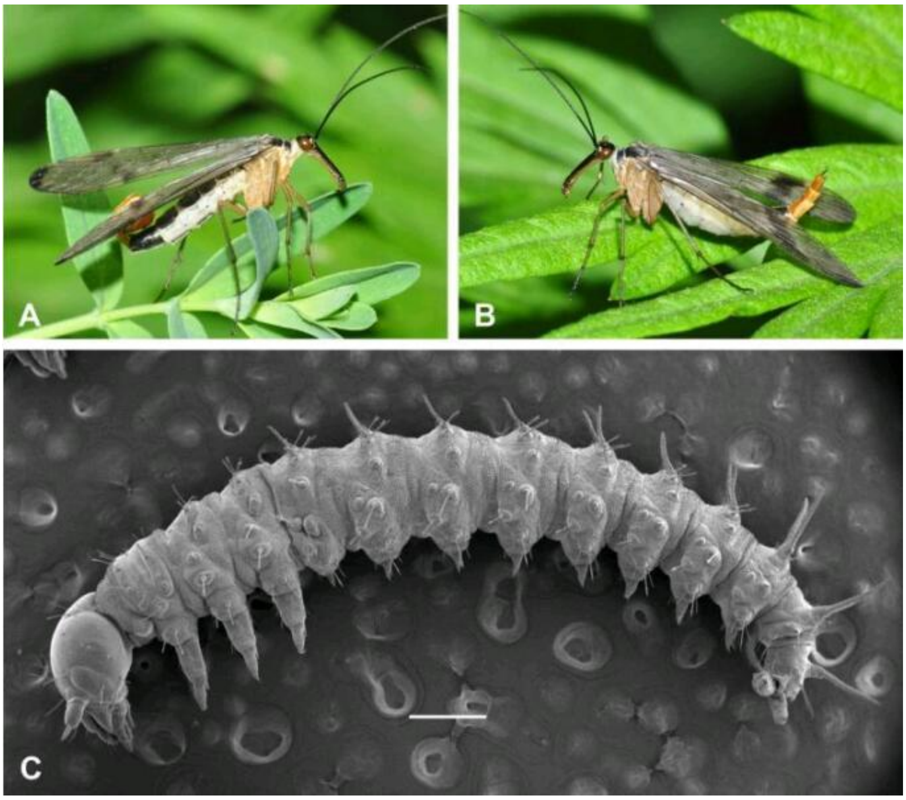

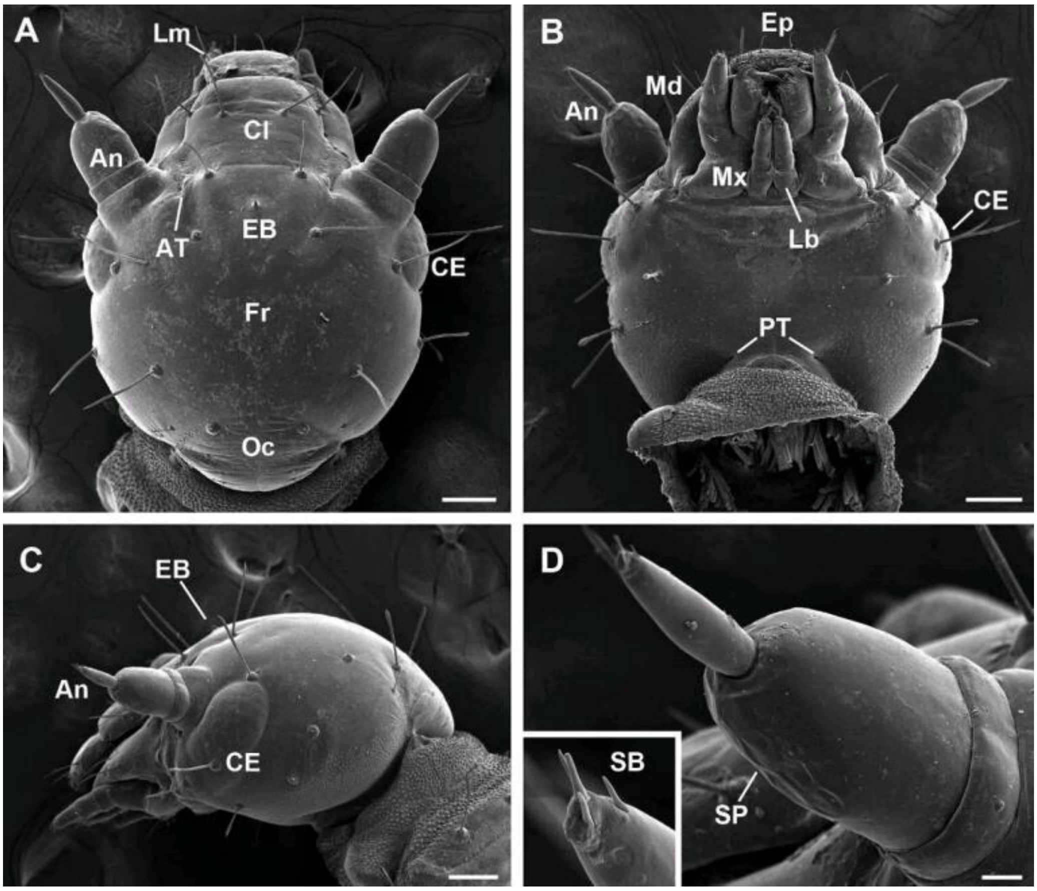

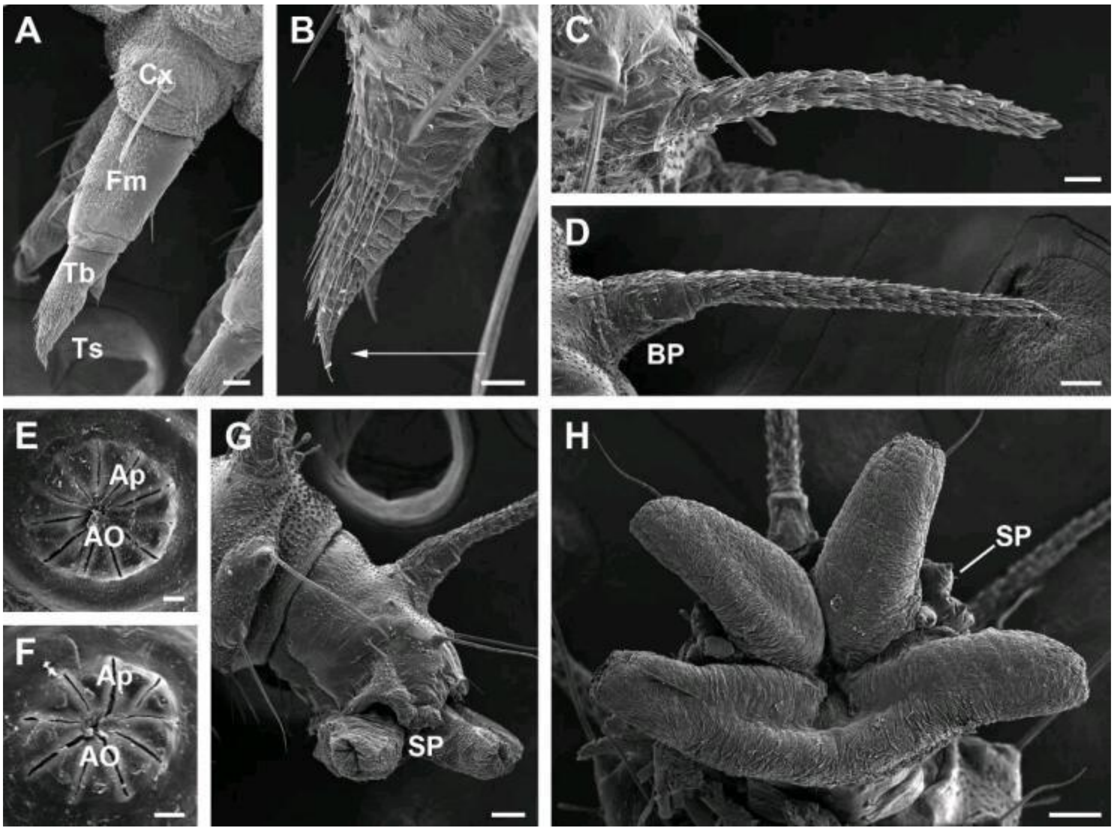
A
Cx
Fm
Tb
Ts
B
C
D
BP
E
Ap
AO
F
Ap
AO
G
SP
H
SP

Panorpa curva

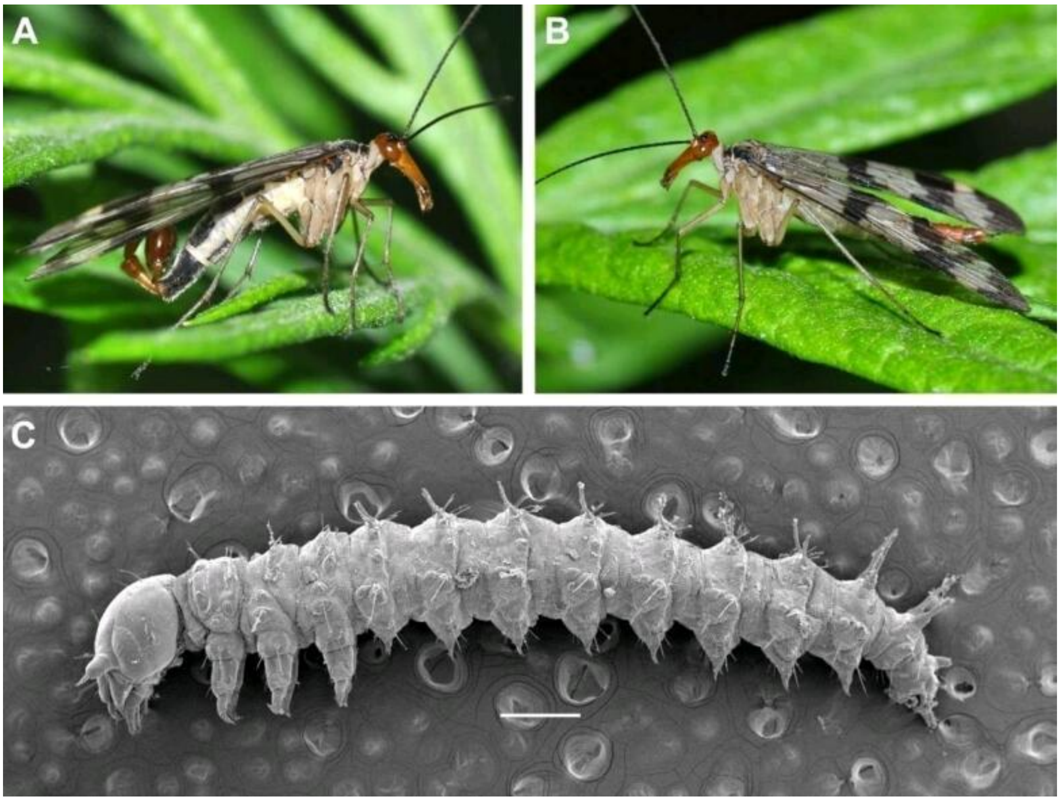

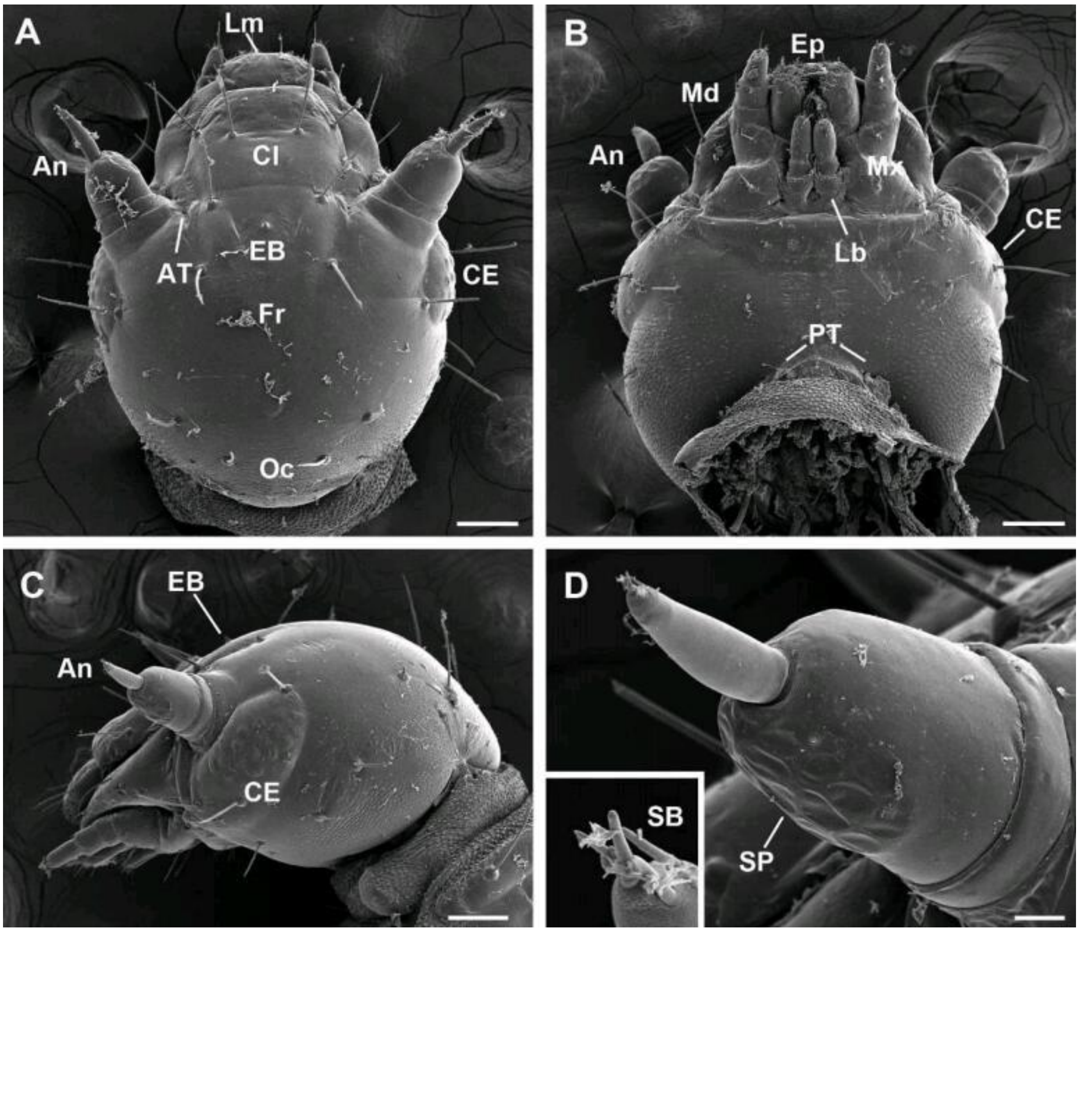

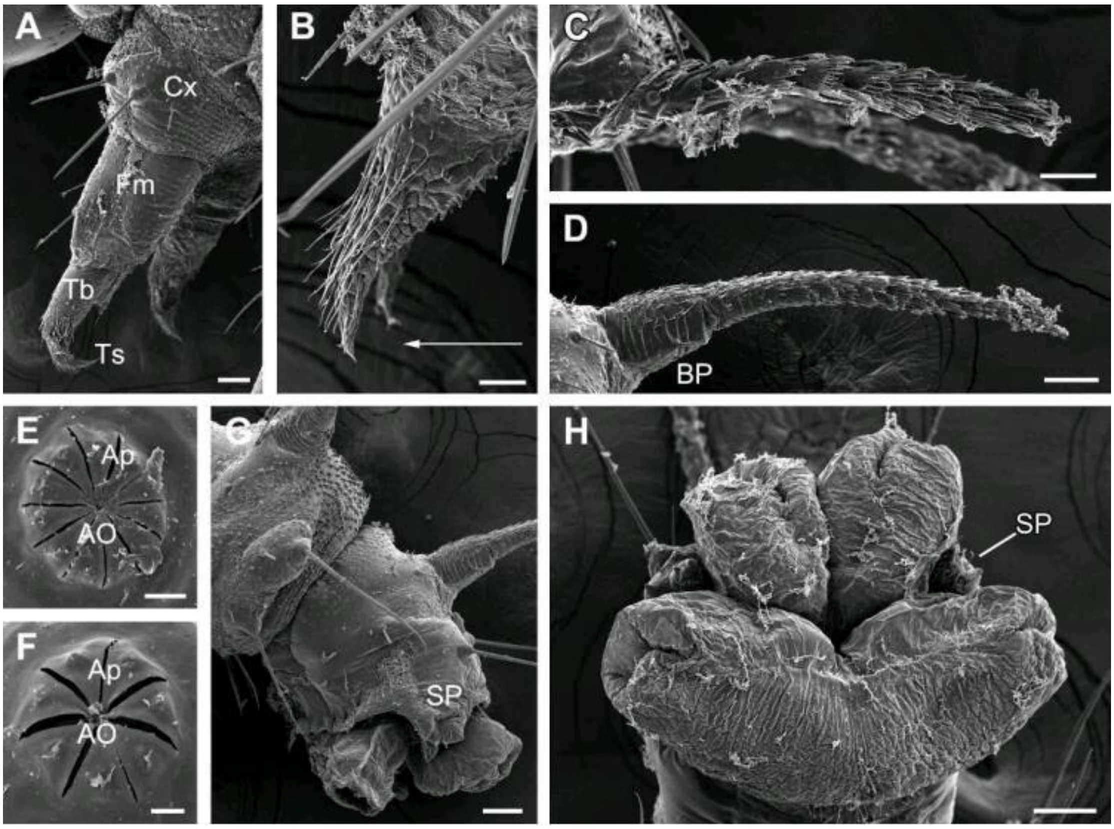

A
Cx
Fm
Tb
Ts
B
C
D
BP
E
Ap
AO
F
Ap
AO
G
SP
H
SP

Panorpa chengi

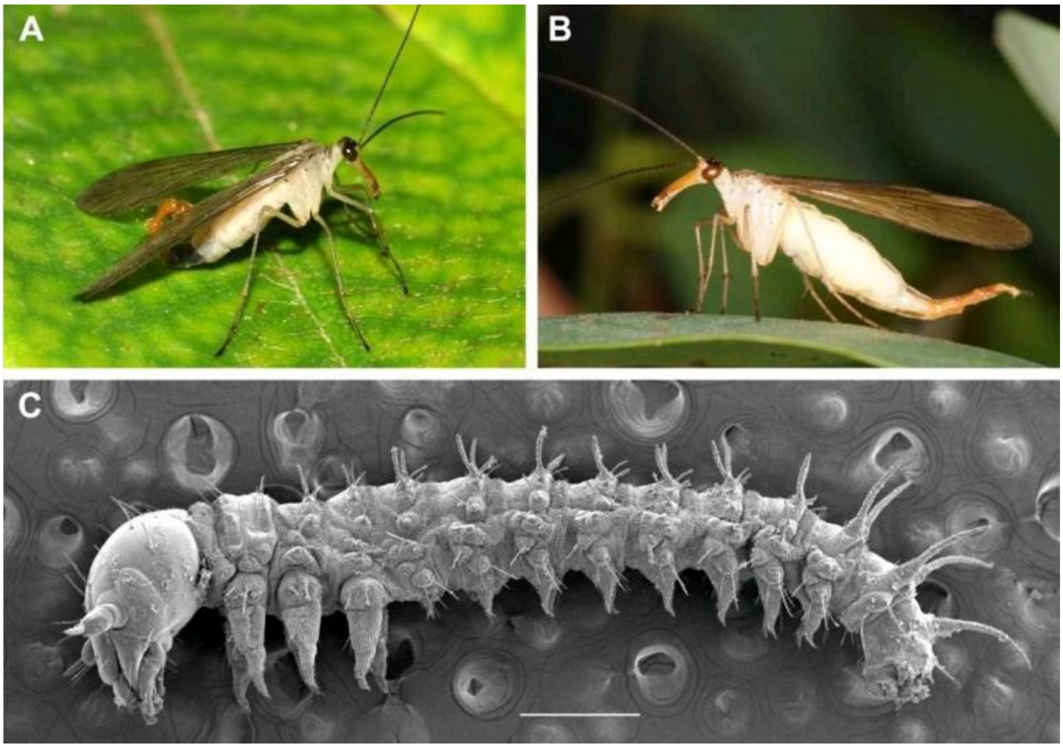

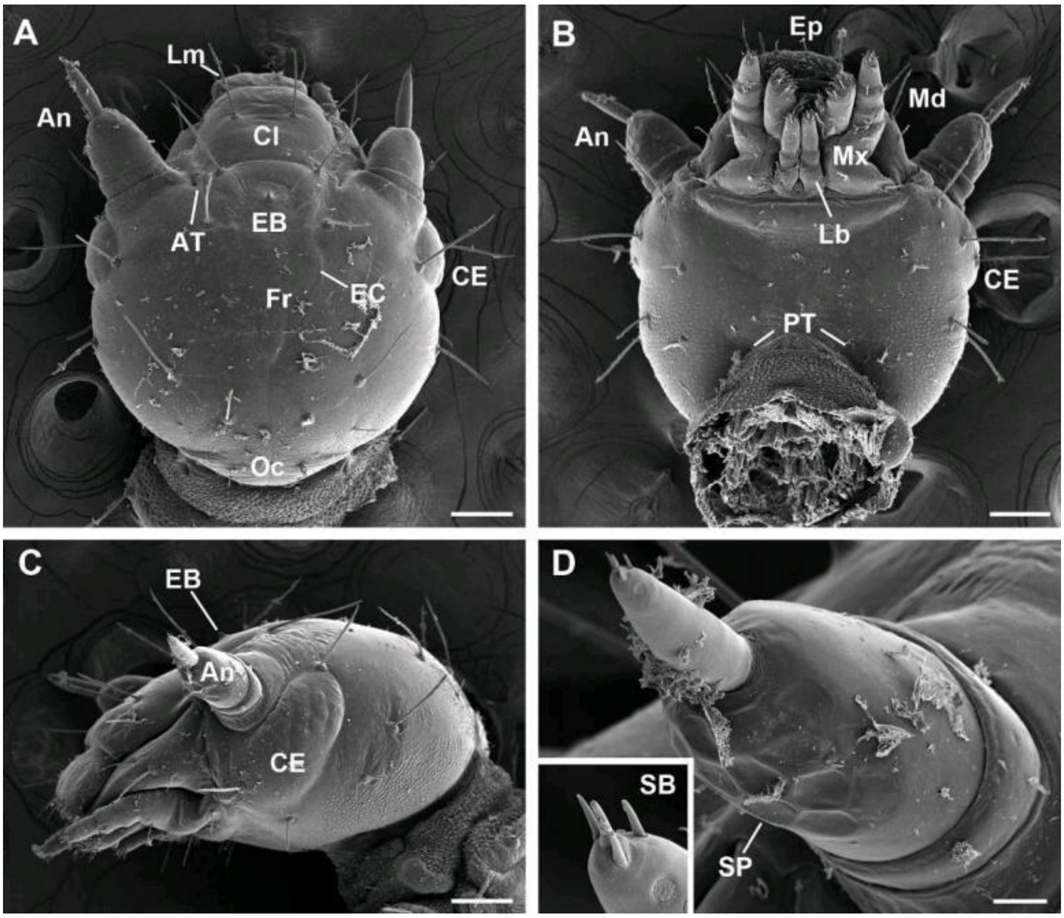

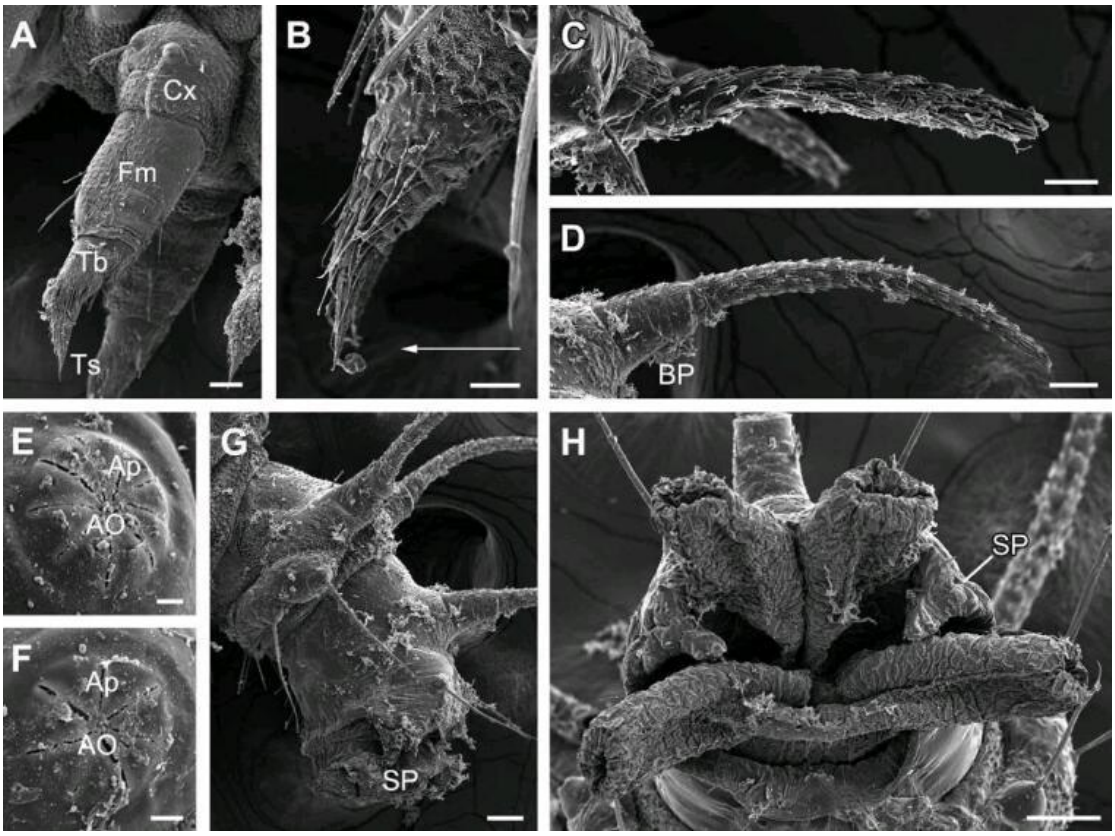
A
Cx
Fm
Tb
Ts
B
C
D
BP
E
Ap
AO
F
Ap
AO
G
SP
H
SP

Neopanorpa lipingensis

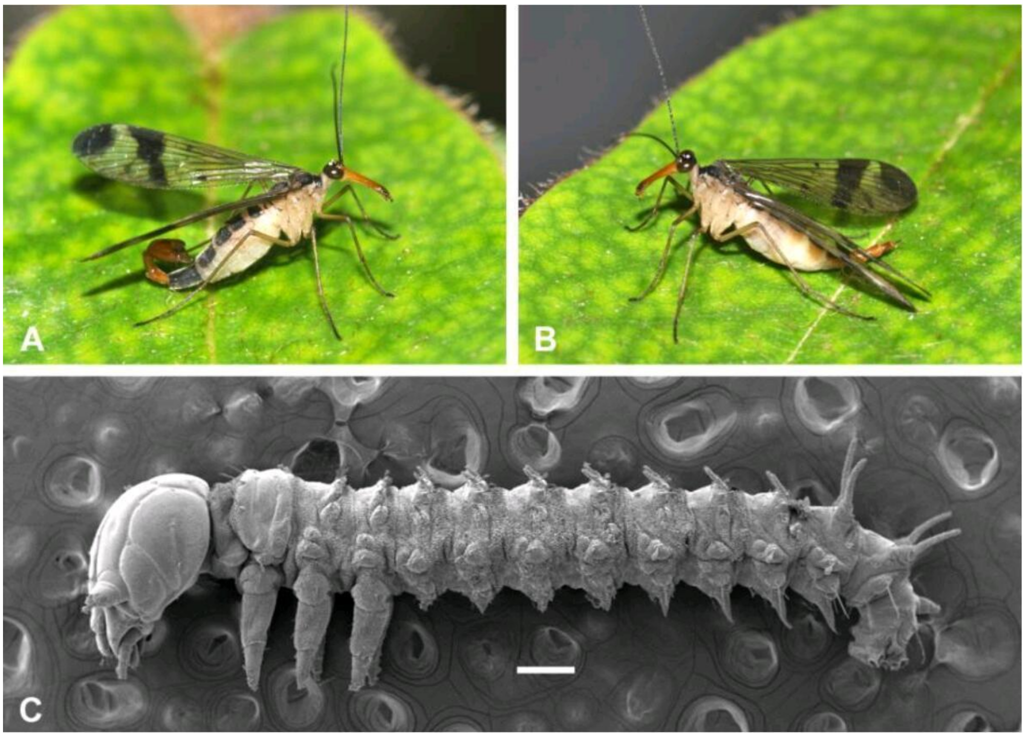

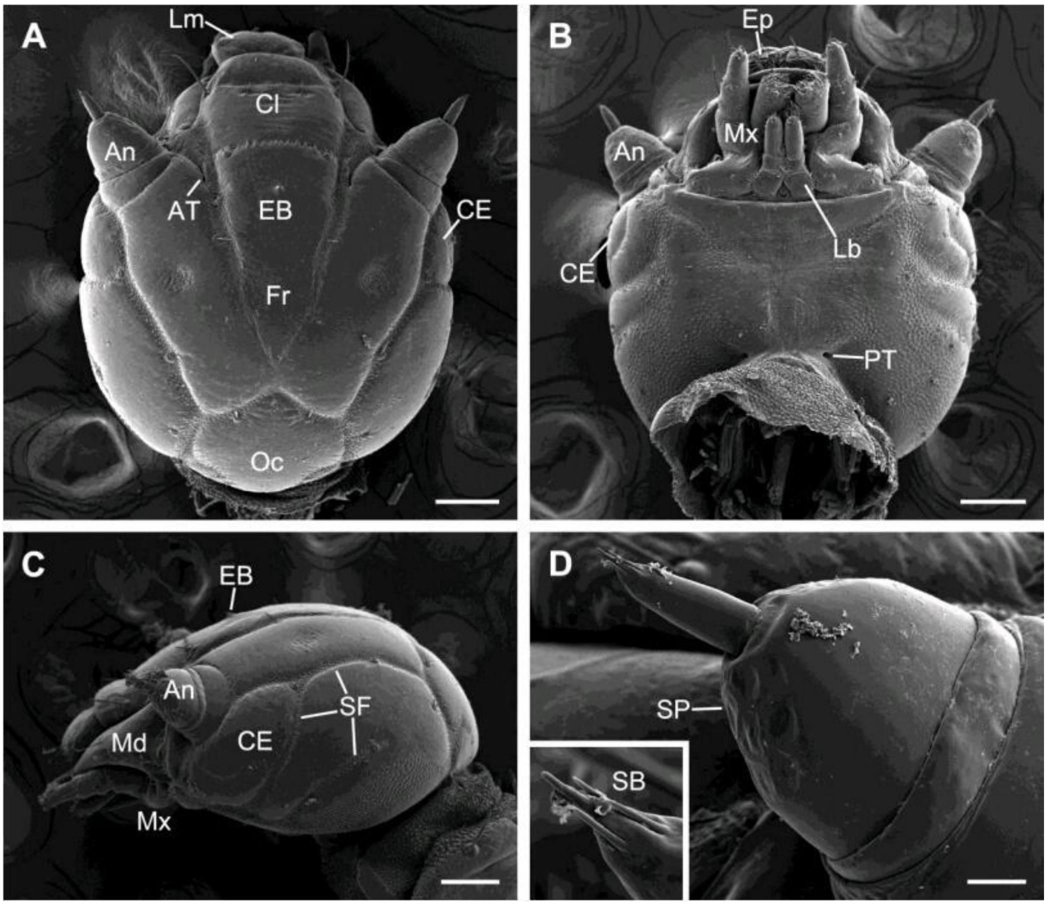

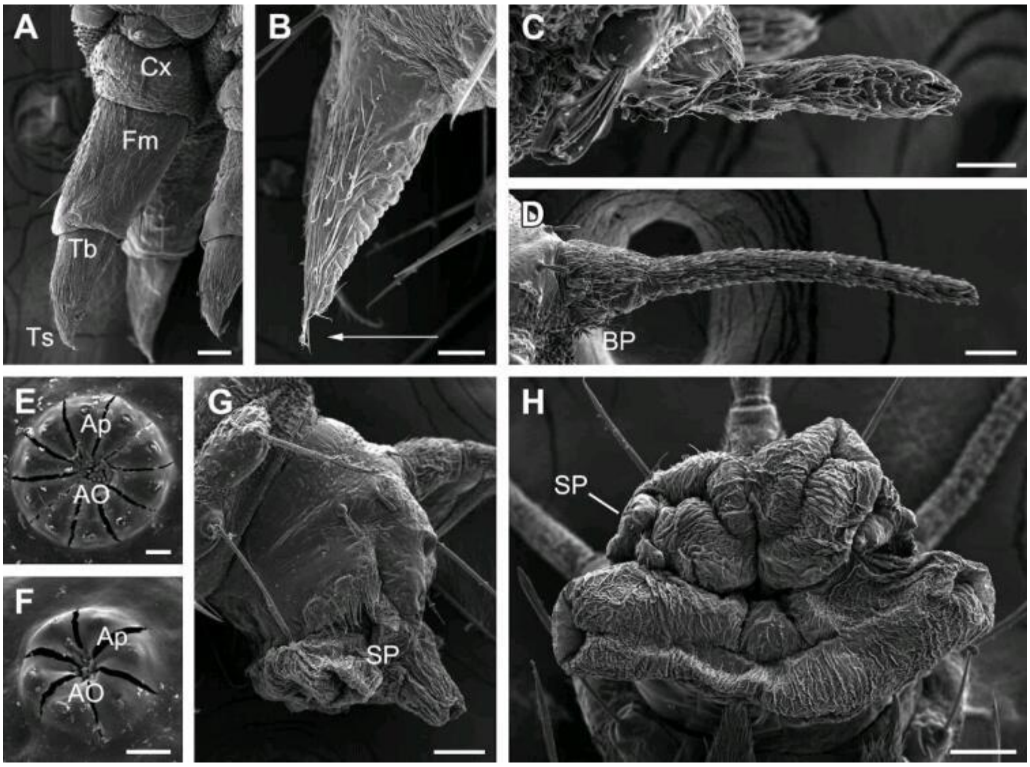

A
Cx
Fm
Tb
Ts
B
C
D
BP
E
Ap
AO
F
Ap
AO
G
SP
H
SP

Neopanorpa longiprocessa

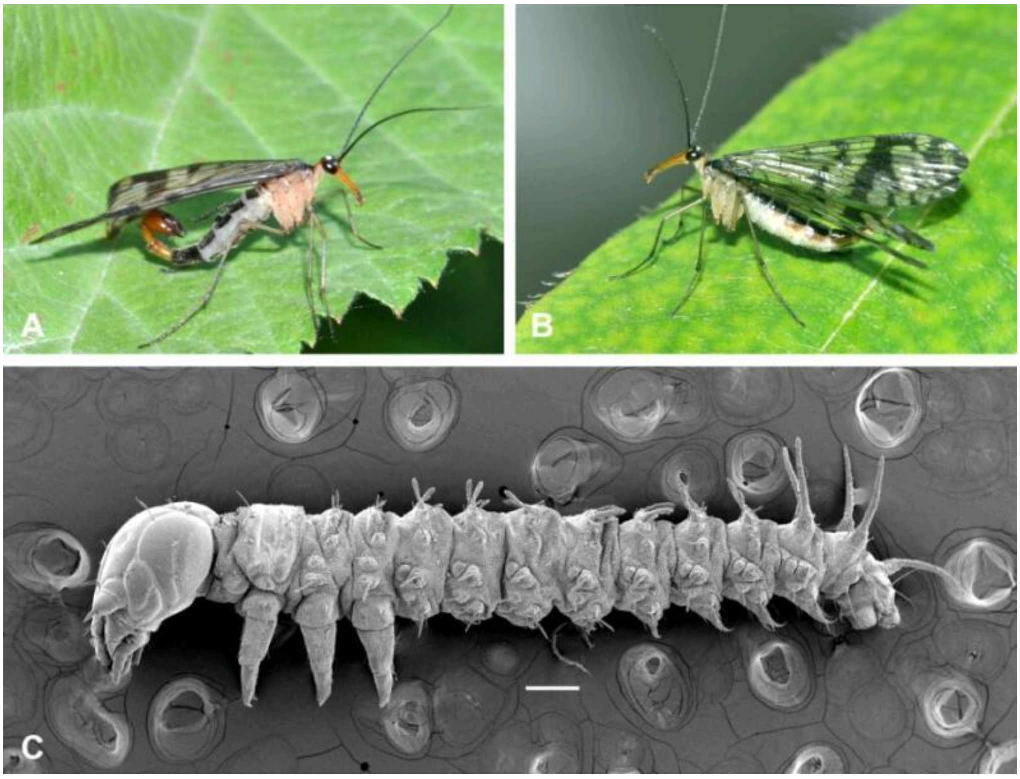

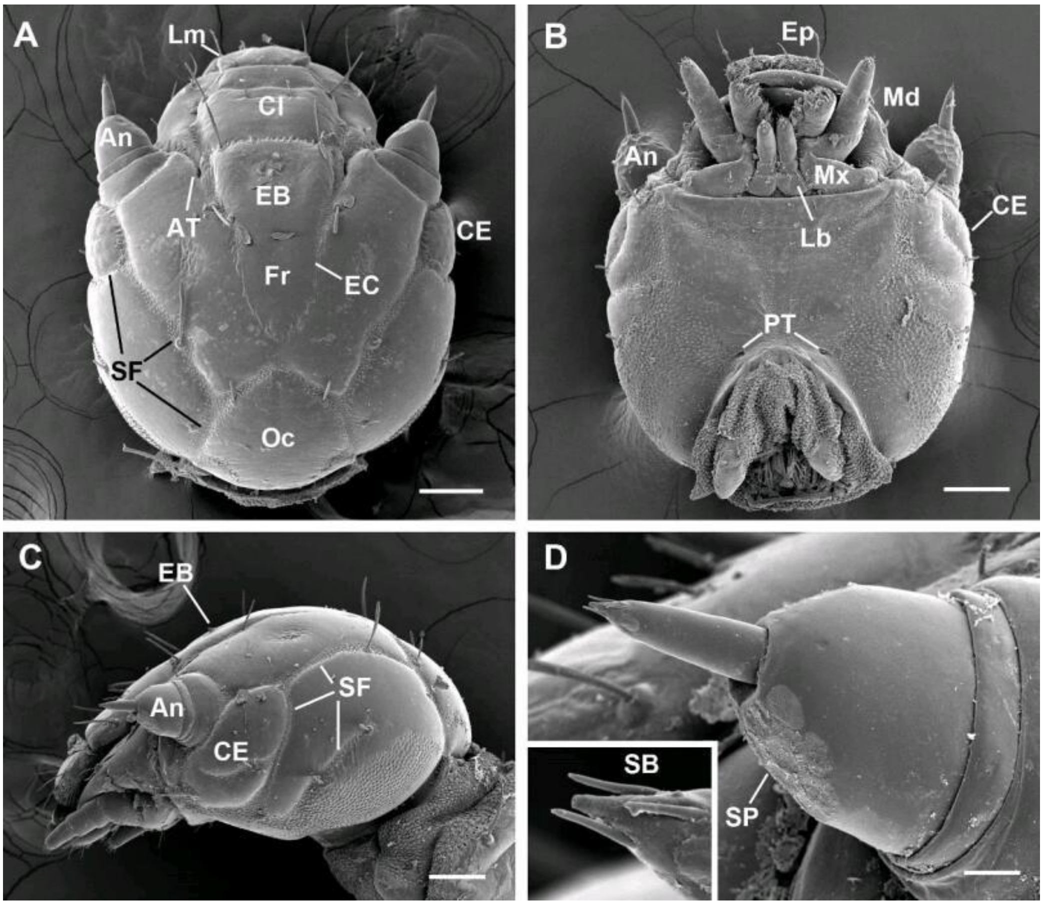

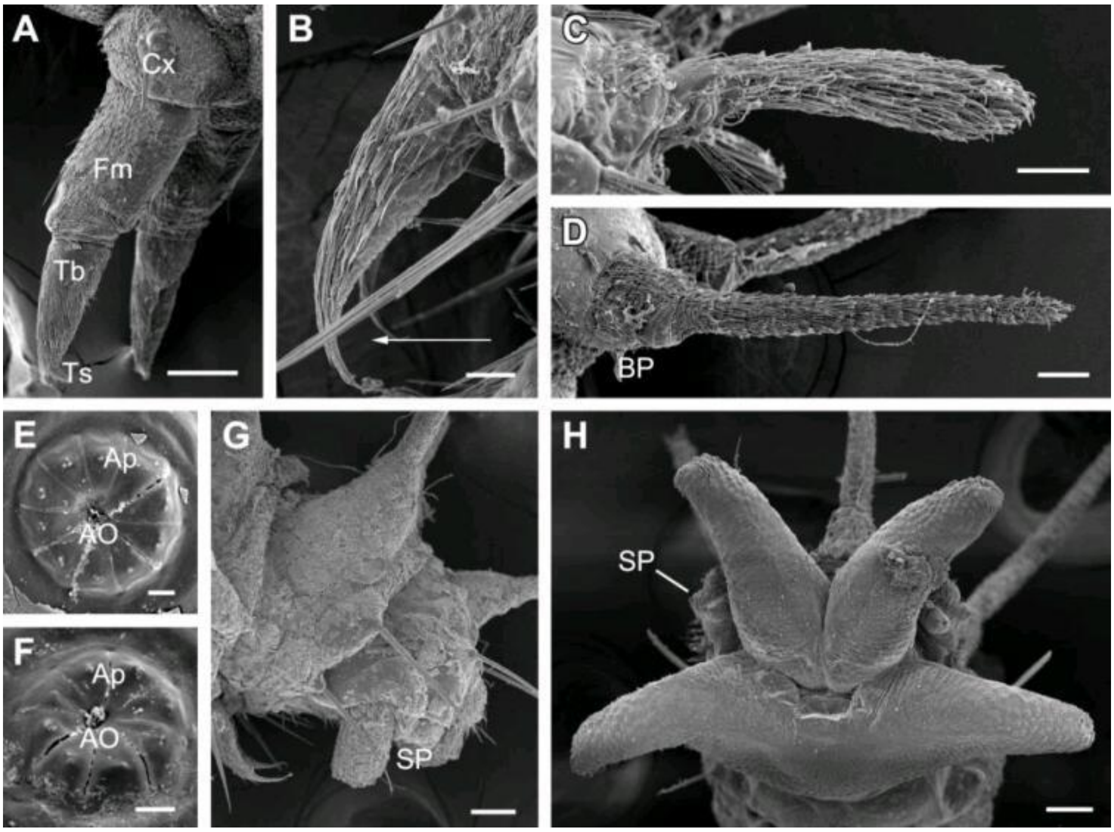

A
Cx
Fm
Tb
Ts
B
C
D
BP
E
Ap
AO
F
Ap
AO
G
SP
H
SP
